# Case report: A fatal case of cryptococcosis in an immunocompetent patient due to *Cryptococcus deuterogattii* (AFLP6/VGII)

**DOI:** 10.1099/jmmcr.0.005168

**Published:** 2018-10-23

**Authors:** M. Bauer, C. Wickenhauser, A. Haak, N. Pazaitis, U. Siebolts, C. Mawrin, C. Strauss, V. Rickerts, D. Stoevesandt, O. A. Cornely, J. F. Meis, F. Hagen

**Affiliations:** ^1^​ Institute of Pathology, Martin-Luther-University Halle-Wittenberg, Halle/Saale, Germany; ^2^​ Institute of Neuropathology, Otto-von-Guericke-University, Magdeburg, Germany; ^3^​ Department of Neurosurgery, Martin-Luther-University Halle-Wittenberg, Halle/Saale, Germany; ^4^​ FG 16, Consultant Laboratory for Cryptococcosis, Scedosporiosis and Imported Systemic Mycoses, Robert-Koch-Institute, Berlin, Germany; ^5^​ Institute of Radiology, Martin-Luther-University Halle-Wittenberg, Halle/Saale, Germany; ^6^​ Cologne Excellence Cluster on Cellular Stress Responses in Aging-Associated Diseases (CECAD), Department I of Internal Medicine, Clinical Trials Centre Cologne (ZKS Köln), University of Cologne, Cologne, Germany; ^7^​ Department Medical Microbiology and Infectious Diseases, Canisius-Wilhelmina Hospital, Nijmegen, The Netherlands; ^8^​ Centre of Expertise in Mycology Radboudumc/CWZ, Nijmegen, The Netherlands; ^9^​ Department of Medical Mycology, Westerdijk Fungal Biodiversity Institute, Utrecht, The Netherlands

**Keywords:** *Cryptococcus gattii*, *Cryptococcus deuterogattii*, endemic areas, pulmonary cryptococcosis, immunocompetent host, cerebral dissemination

## Abstract

**Introduction:**

Cryptococcosis in immunocompetent adults is a rare disease in Europe, mostly induced by members of the *Cryptococcus gattii* species complex. The diagnosis can be challenging due to its rarity, unspecific symptoms and long symptomless latency.

**Case presentation:**

A 49-year-old woman with a three weeks history of headache was admitted to the hospital due to discrete ataxia and impaired vision. Cranial magnetic resonance imaging (MRI) showed a contrast-enhancing mass in the cerebellum. Further investigations detected a slight leukocytosis and a single subpleural nodule in the right inferior lung lobe. The cerebral lesion was surgically removed, and a direct frozen section only showed an unspecific inflammation. In the course of her admission she developed non-treatable cerebral edema and died ten days after surgical intervention. Histopathological examination of the surgical specimen and postmortem evaluation of the lung and the cerebrum demonstrated fungal elements. Molecular identification of the fungal elements in formalin-fixed paraffin-embedded tissue lead to the diagnosis of cryptococcosis induced by *C. gattii sensu lato*. Molecular genetic analysis identified the involved cryptococcal species as genotype AFLP6/VGII, recently described as *Cryptococcus deuterogattii*, which is known to be endemic to the west-coast of Canada and the USA. Additional heteroanamnestic information revealed that she had spent her holidays on Vancouver Island, Canada, two years before disease onset, indicating that infection during this stay seems to be plausible.

**Conclusion:**

Cryptococcosis due to *C. deuterogattii* is a rarely encountered fungal disease in Europe, not particularly associated with immunodeficiency, and infection is likely to be contracted in endemic areas. Due to its rarity, long symptomless latency, unspecific symptoms and misleading radiological features the diagnosis can be challenging. Physicians need to be aware of this differential diagnosis in immunocompetent patients, as early adequate therapy can be lifesaving.

## Introduction

In Western Europe *Cryptococcus gattii* infections have increased during the last two decades [1]. *C. gattii* can cause severe systemic disease in immunocompromised and immunocompetent hosts [2–4]. *Cryptococcal* yeasts usually enter the host via the respiratory tract [2]. Pulmonary symptoms as well as radiographic features and clinical parameters are mostly unspecific and often lead to misdiagnosis [5]. Both *Cryptococcus neoformans* and *C. gattii* species complexes show a high affinity for the central nervous system [6]. The median incubation time is six to seven months [7, 8]. By using molecular techniques, such as restriction fragment length polymorphism (RFLP), PCR fingerprinting, multi-locus sequence typing (MLST) and amplified fragment length polymorphism (AFLP) fingerprinting it was observed that *C. gattii* species complex can be differentiated into five distinct genotypes [1]. Due to epidemiological, genetic and ecological differences the taxonomy of the *C. neoformans*/*C. gattii* species complexes have been recently thoroughly revised and the five *C. gattii* species complex genotypes were all raised to species as *C. gattii sensu stricto* (genotype AFLP4/VGI), *Cryptococcus bacillisporus* (genotype AFLP5/VGIII), *Cryptococcus deuterogattii* (genotype AFLP6/VGII), *Cryptococcus tetragattii* (genotype AFLP7/VGIV) and *Cryptococcus decagattii* (genotype AFLP10) [9]. The globally occurring species *Cryptococcus gattii s.s.* and *C. deuterogattii* are often involved in disease in apparently immunocompetent subjects, while the other three species are geographically restricted and have a predilection for immunocompromised hosts, mostly HIV-infected individuals [9]. An ongoing outbreak in British Columbia (Canada) that was first recognized in 1999 and started on Vancouver Island, was found to be caused by the rare genotype AFLP6/VGII (=*C. deuterogattii*). The outbreak expanded to the Pacific Northwest of the USA, although different subgenotypes were involved [10, 11] and has affected the health of hundreds of humans, and many more animals living in that area [12, 13]. In Germany, less than 25 hospitalizations for cryptococcosis are listed per year with 3 % documented *C. gattii s.l.* infections. As molecular analysis identified that only half of the infections were acquired abroad [1], identification of environmental niches occupied by *C. gattii s.l.* in Germany is an important challenge to assess the associated risk of infection [14]. Here we report a fatal case of cryptococcosis due to *C. deuterogattii* infection that was likely to have been acquired on Vancouver Island two years before the onset of disease.

## Case report

**Fig. 1.**
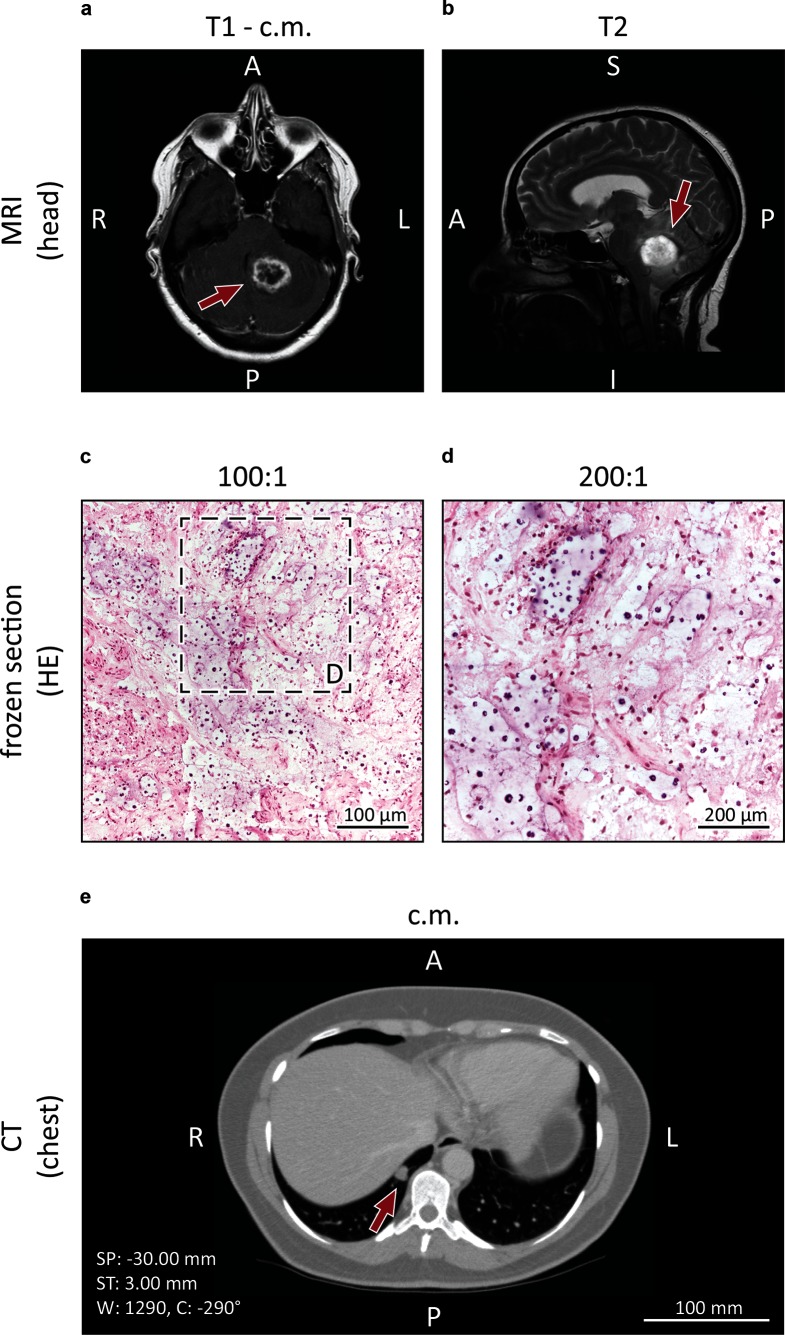
Preoperative and intraoperative diagnostic investigations. Cranial magnetic resonance imaging (a and b) showing a circumscribed mass in the left cerebellum hemisphere (arrows). Further investigations included a chest computed tomography (e) with presentation of a subpleural nodule in the right lower lobe (arrow). An intraoperative frozen section (c and d) revealed paucicellular glial tissue with several infiltrating immune cells.

**Fig. 2.**
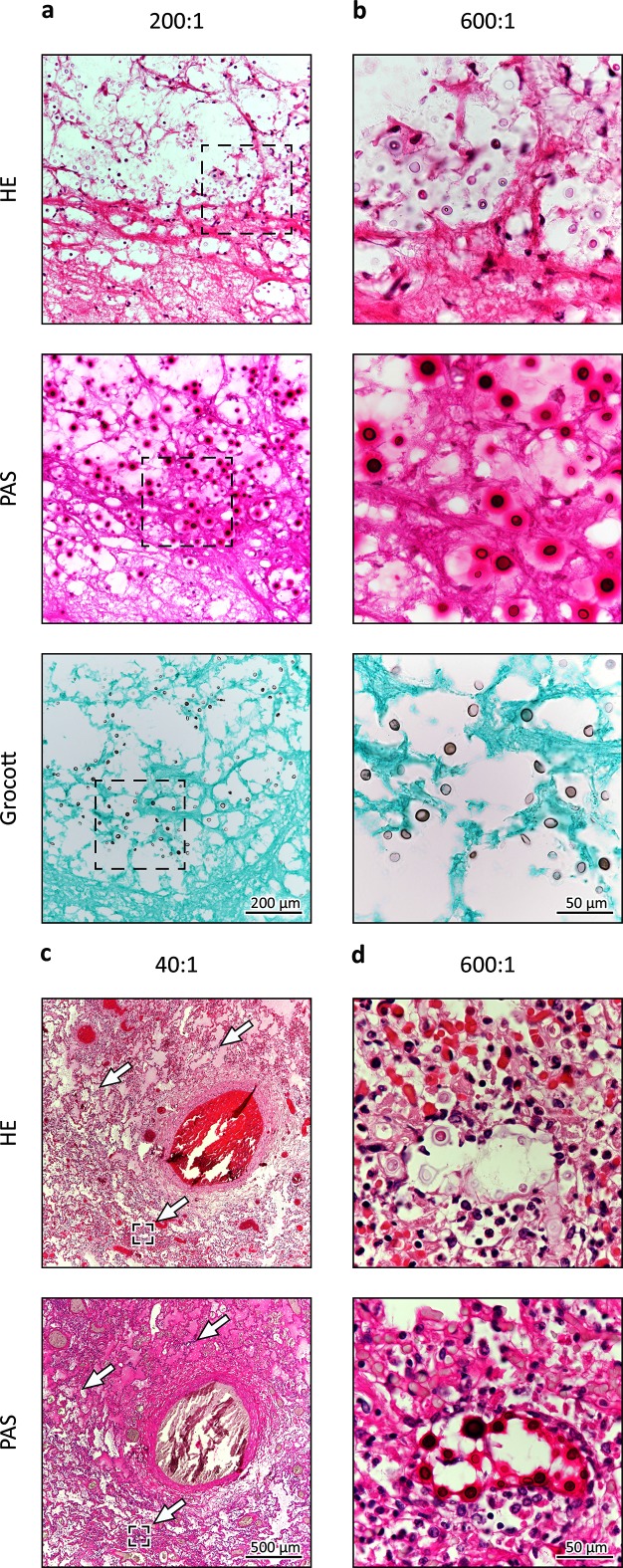
Postmortem diagnostic investigations. Cerebellar tissue (a and b) showing single immune cells next to some poorly demarcating spherules in HE-staining. Assessment of lung tissue (c and d) revealed a purulent pneumonia with presentation of several foci with the same round and oval structures (Arrows). Further staining of the cerebellar tissue as well as of the lung tissue including PAS reaction and Grocott staining supported the assumption that this was due to *Cryptococcus*.

**Fig. 3.**
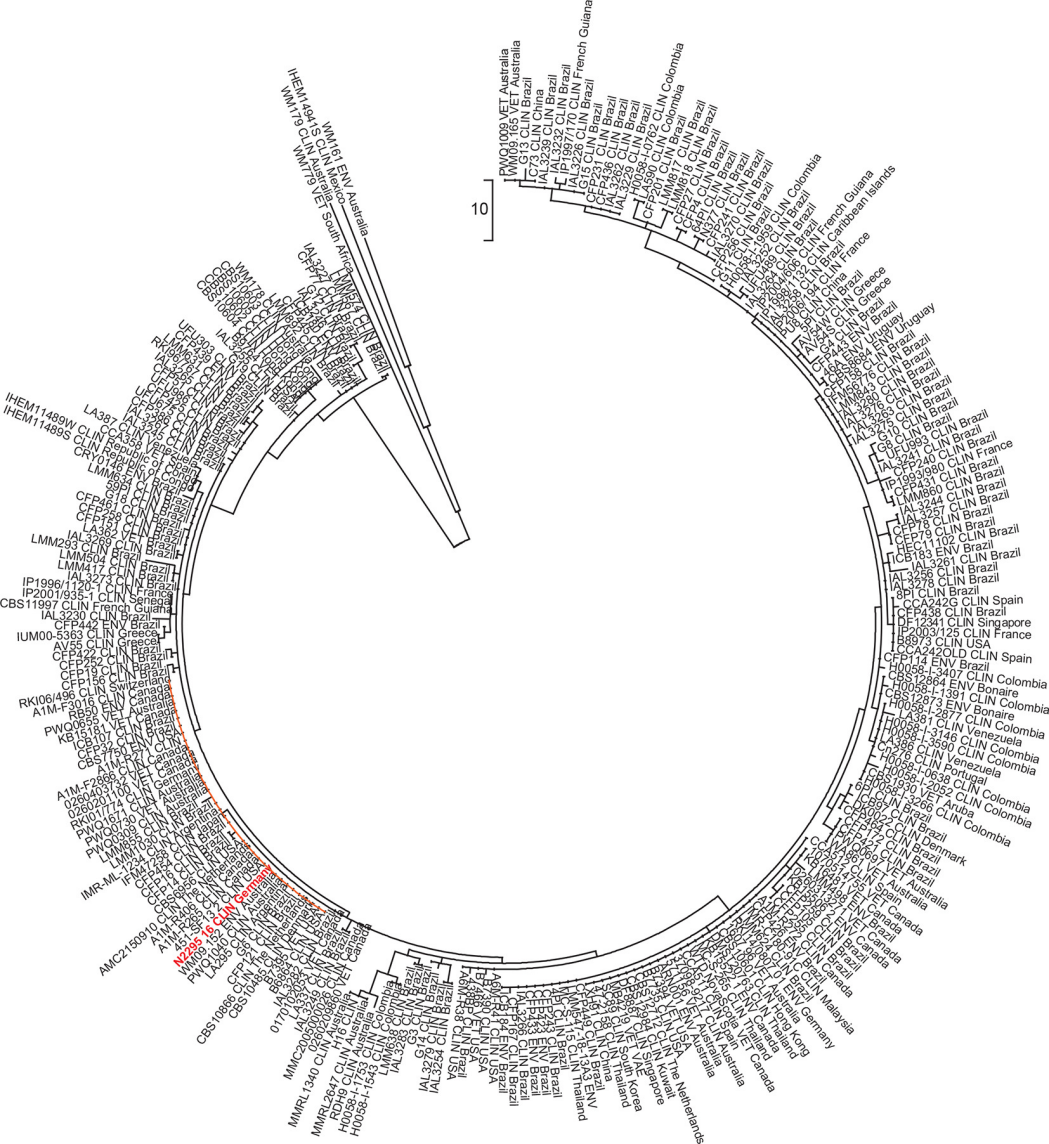
Phylogenetic analysis Using phylogenetic analysis, it was observed that this strain clustered within the clade of Vancouver-Island-outbreak-related *C. deuterogattii* strains.

A 49-year-old woman was hospitalized in the University Hospital Halle/Saale, Germany, with an acute presentation of headache, discrete ataxia and impaired vision. No episodes of pyrexia were reported. Serum inflammatory markers were inconspicuous with only a slight leukocytosis (14.53 Gpt l^−1^). Cranial magnetic resonance imaging (MRI) revealed a contrast-enhancing lesion with surrounding edema in the left cerebellum hemisphere next to the cerebellar pedunculus (Fig. 1a, b). In addition, computed tomography (CT) showed a small hyperintense subpleural nodule in the right lower lung lobe (Fig. 1e). To rule out malignancy the cerebellar lesion was surgically biopsied via a suboccipital craniotomy. A frozen section contained paucicellular glial tissue without signs of malignancy, purulence or specific infection (Fig. 1c, d). Postoperatively, the patient developed cerebral edema with displacement and compression of the fourth ventricle and the brainstem. Severe increase of intracranial pressure required suboccipital craniotomy and application of cerebrospinal fluid (CSF) drainage. A microbiological examination of the liquor was not carried out as there was no suspicion of an infection. Despite extensive supportive care the clinical condition did not improve and ten days after hospitalization the patient died. An autopsy demonstrated a purulent pneumonia with punctum maximum in the right inferior lung lobe. In addition, further processing of the cerebral biopsy was undertaken, including PAS and Grocott staining. These revealed the image of cerebral cryptococcosis with presentation of typical capsule, highlighted fungi next to a surrounding histiocyte-rich inflammation with abundance of foam cells and a rare lymphocytic infiltrate (Fig. 2a, b). The same pathogens were also seen in the inflamed pulmonary tissue (Fig. 2c, d). Cryptococcal DNA was amplified by a broad-range PCR assay targeting the ITS2 region of the ribosomal DNA. The amplicon was identified as *C. gattii* by LCD-Chip hybridization (LCD array fungi 2.1; Chipron). Natural reservoirs for *C. gattii* are found in Australia, Asia, Africa and some regions of America, including Vancouver Island which has been determined to be an endemic area [1]. In contrast, infection in Europe is rare, although *C. gattii* has been previously isolated in Greece, southern Italy and Spain [15]. When confronted with the autopsy results the relatives of the deceased mentioned that she spent her holidays on Vancouver Island two years prior her death. Tissue samples were transmitted to the Robert-Koch Institute (Berlin, Germany) where molecular pathogen analysis was confirmed by amplification of the IGS region of the ribosomal DNA with amplicon detection by LCD-Chip hybridization using a commercial system (Chipron) and ITS2 PCR with amplicon sequencing [16]. Subsequently in Nijmegen, the Netherlands, a standard multi-locus sequence typing analysis was performed [1], which did not yield any PCR product due to the fragmented formalin-fixed paraffin-embedded (FFPE)-DNA. Therefore a FFPE-MLST scheme was developed to cover the most variable parts of each of the seven standard MLST-loci (Table 1). PCRs and sequencing were performed as described previously [1]. Using the FFPE-MLST it was determined that the involved cryptococcal strain was *C. deuterogattii*, formerly known as *C. gattii* genotype AFLP6/VGII. By using phylogenetic analysis, it was determined that this strain clustered within the clade of Vancouver-Island-outbreak-related *C. deuterogattii* strains (Fig. 3), which supports the earlier suggestion that the patient acquired the cryptococcal infection during her holiday to Vancouver Island.

**Table 1.** Newly designed *Cryptococcus gattii sensu lato* FFPE-MLST primers

Locus	Primer name	Primer sequence*
CAP59	FFPE-CAP59Fwd	AGGCGAGGCAGCACAAGTA
	FFPE-CAP59Rvd	TTTGTCTGGTCGTTGGAACC
GPD1	FFPE-GPD1Fwd	AGGTCGTATCGGTCGAATTG
	FFPE-GPD1Rvd	CCATGTAGTCCAAGTCAATGAAA
IGS1	FFPE-IGS1Fwd	TTGGCTAAGATGCGTTATGC
	FFPE-IGS1Rvd	TTGCTTGACCGAGCTTGACT
LAC1	FFPE-LAC1Fwd	CATGGTATGCGGCAGAAG
	FFPE-LAC1Rvd	AAGCCTATGGTACSTCATCAGC
PLB1	FFPE-PLB1Fwd	CGTGGATTAGAAATGCCACTGT
	FFPE-PLB1Rvd	TTCGGTGCTTTCATTCATCA
SOD1	FFPE-SOD1Fwd	ACTCTGAGAGGCACGTTGGT
	FFPE-SOD1Rvd	TGATGGAGTAAGGGCCAAAG
URA5	FFPE-URA5Fwd	AGGCCGGTGAGCCATATC
	FFPE-URA5Rvd	CGTCCTTCTTCTCCTTCCTG

*All primers have a Tm of 60 °C, PCRs were performed as described previously [1].

## Discussion

*Cryptococcus* is a common yeast fungus with a natural reservoir on decaying wood and bird droppings [12–14]. Until recently, the genus *Cryptococcus* included over 100 species, but after a thorough taxonomic revision only ten remained [9]. Members of the *C. gattii* species complex, which is common in Sub-Saharan Africa, Asia, Australia, Europe, South and North America, can infect persons without immunodeficiency [2, 12, 13, 15–17]. As a diagnostic challenge, the patients do not present typical clinical or laboratory findings. Furthermore, it has been reported that the infection first involves the pulmonary tract and spreads secondarily to organs, especially the central nervous system [18]. Frequently, the patients present with a respiratory syndrome with cough, dyspnea and chest pain while neurological symptoms, like headache and neck stiffness, as well as night sweats, weight loss and anorexia may occur afterwards [2]. Additionally, it has been reported that the clinical symptoms and the progression of the disease differ between species within the *C. gattii* species complex [12, 18–20], even *in vitro* antifungal susceptibility varies [21]. Concerning the case presented here, a woman with cough over a period longer than one year suddenly developed acute headache, discrete ataxia and impaired vision. Initially, the blurred mass in the cerebellum together with the subpleural nodule in the right lower lung lobe was misinterpreted as malignancy, a finding that is described in several other reports [5, 13, 18, 21–25]. The hypothesis of malignancy was further supported by clinical and laboratory findings, as the patient had no fever and only mild leukocytosis. Finally, the diagnosis of cryptococcosis was only made post mortem after histopathological and molecular-pathological evaluation. A main reason for the delayed diagnosis is the lack of experience due to the low incidence in an unexpected patient group. It should also be noted that the time between infection and the first clinical symptoms was uncommonly long, compared with a reported median incubation time of between six and seven months [7, 8]. According to data from the Robert Koch Institute in Berlin, Germany, only three percent of 155 cases of cryptococcosis were related to *C gattii s.l.* between 2004 and 2013 [13, 14] and surveillance is challenging because fungal infections are not notifiable diseases. The available data indicate that there was no significant increase of cryptococcosis during that period [14]. However, in terms of health policy it is meaningful that *C. gattii s.l.* infections might also occur in Northern Europe, probably as a consequence of climate change [14, 26]. Therefore, nowadays this fungal pathogen is not only restricted to tropical and subtropical regions [14] and endemic areas for *C. gattii s.l.* within Europe have been described [1]. In 2012 the fungus was isolated from a tree in the Netherlands [27] and earlier *C. gattii s.l.* was detected in Italy, Greece, Portugal, and Spain [1, 15, 21, 28]. Furthermore, in Germany few cases of cryptococcosis due to *C. gattii s.s.* without a travel history to endemic regions have been documented [14]. Nonetheless at least half of the infections in Germany were acquired abroad [1, 14]. In summary, it is important that clinicians are aware of this challenging invasive mycosis that often infects immunocompetent subjects. Unspecific symptoms with cough in conjunction with acute cerebral symptoms are indicators for the diagnosis and it has to be pointed out that an early diagnosis allows cure of the majority of patients, albeit that despite effective treatment central nervous complications may persist [6].

### Conclusion

In Western Europe, increased international travel leads to a rising number of infections with rare pathogens [29]. Additionally climate change creates new ecological niches in the environment. *C. gattii*, which has been formerly known to be a pathogen in tropical and subtropical regions [2, 3], has been recognized in temperate regions in Europe and North America [1, 10, 15]. In consequence of the lack of experience due to the low incidence in a particularly unexpected group of patients, determination of the diagnosis is often delayed and findings are misinterpreted [13, 14]. Early diagnosis is critical for a successful treatment of this otherwise lethal infection mostly due to central nervous complications [6].
